# CLIMATE CHANGE POLICIES AND OLDER ADULTS: AN ANALYSIS OF STATES’ CLIMATE ADAPTATION PLANS

**DOI:** 10.1093/geroni/igad104.0502

**Published:** 2023-12-21

**Authors:** Jacklyn Kohon, Bryant Carlson, Paula Carder, Dani Himes, Eiji Toda, Katsuya Tanaka

**Affiliations:** Portland State University, Portland, Oregon, United States; Portland State University, Portland, Oregon, United States; Portland State University Institute on Aging, Portland, Oregon, United States; Institute on Aging, Portland, Oregon, United States; Portland State University, Portland, Oregon, United States; Shiga University, Hikone, Shiga, Japan

## Abstract

As climate change drives more frequent and intense weather events, older adults face disproportionate impacts, including having the highest mortality rates from storms, wildfires, flooding, and heat waves. State governments are critical in deploying local resources to help address climate change impacts. This policy study analyzes states’ climate adaptation plans to assess the methods through which they address the impact of climate change on older adults. This study uses content analysis to analyze available climate change adaptation plans for all U.S. states for strategies designed to increase resilience of older adults to impacts of climate change. Nineteen states have climate adaptation plans, of which 18 describe older adults as a population group with specific health impacts and risks factors. Four categories of adaptation strategies for older adults include communications, transportation, housing and emergency services. State plans vary in terms of the risk factors and adaptation strategies included. To varying degrees, states’ climate change adaptation planning address health, social and economic risks specific to older adults, as well as strategies for mitigating those risks. As global warming continues, collaborations between public and private sectors and across regions will be needed to prevent negative outcomes such as forced relocation and other social and economic disruptions as well as disparate morbidity and mortality.

